# The Influence of Framing Plant-Based Products in Terms of Their Health vs. Environmental Benefits: Interactions with Individual Wellbeing

**DOI:** 10.3390/ijerph191911948

**Published:** 2022-09-21

**Authors:** Amy Isham, Judith Geusen, Birgitta Gatersleben

**Affiliations:** 1 Centre for the Understanding of Sustainable Prosperity (CUSP), University of Surrey, Guildford GU2 7XH, UK; 2 School of Psychology, University of Surrey, Guildford GU2 7XH, UK

**Keywords:** plant-based foods, food labelling, message framing, wellbeing, willingness to pay, product liking

## Abstract

Significant reductions in the consumption of meat and dairy products are required to limit environmental damage and meet climate targets. However, individuals choosing to adopt plant-based diets still form a minority. Whilst different types of message framings have been suggested to be a potential means of encouraging the uptake of plant-based diets, recent findings have often failed to document any differences in people’s willingness to reduce their consumption of animal products based on whether messages emphasize the health or environmental benefits of plant-based diets. This research examined whether individual wellbeing might interact with health versus environmental message frames to determine people’s liking and willingness to pay for plant-based products. Across two experiments with a university (Study 1) and a non-student, adult sample (Study 2), participants were exposed to different hypothetical labels for plant-based foods and asked to rate their liking and willingness to pay for each. In line with existing findings, results demonstrated a trend whereby showing health (versus environmental) information on food labels did not in itself influence participants perceptions of the food products. Higher levels of positive wellbeing were associated with greater liking and willingness to pay for the plant-based foods (B values ranging from 0.04 to 0.45). Further, there was an interaction effect whereby levels of negative affect were differentially linked to liking and willingness to pay across the health and environmental framing conditions (B values ranging from 0.03 to 0.38). In particular, negative affect appears to have a greater negative impact on the product liking and willingness to pay when environmental label framings are used. This effect was most pronounced for the product liking dependent variable (B = −0.29 in the environmental framing condition). This research therefore extends understandings of the more specific instances in which message framings can impact perceptions of plant-based foods. The implications of the findings for understanding how best to promote uptake of plant-based diets are discussed.

## 1. Introduction

Significant reductions in meat and dairy consumption have been proposed as critical for limiting environmental impacts [1,2]. It is estimated that livestock contributes roughly 5% of the total CO_2_ emissions caused by human activities each year [3], whilst also being the largest cause of human-induced methane emissions [4]. However, the production of animal products does not only have negative impacts in terms of rising greenhouse gas emissions. It also demands water use, reduces water quality, erodes soil, and leads to loss of animal habitats and biodiversity as areas are deforested to create pasture or arable land for animal feed [3,5]. At the same time, high consumption of meat products (especially processed or red meat) has been linked to adverse health outcomes such as increased risk of mortality and type 2 diabetes [6].

The EAT Lancet Commission proposes a “planetary health diet” as one which contains only modest, and optional, amounts of animal sources of protein [7]. Plant-based diets can comprise fruit, vegetable, wholegrains, and legumes, as well as more direct substitutes such as cultured meat or nut-based milks. The production and consumption of plant-based foods accounts for only half as many greenhouse gas emissions as does the production and consumption of animal-based foods [8]. If we compare the production of beef and beans as an example, the same amount of protein can be produced in beans using over ten times less land, water, fertilizers, and pesticides in comparison to producing the same amount of protein in beef [9,10]. Despite this, those people who chose to adopt a plant-based diet still make up only a minority of consumers [11]. It is therefore crucial that we better understand the factors that encourage or discourage people to consume plant-based products, to be able to prompt this form of sustainable behaviour change on a larger scale.

This research explores how people’s wellbeing and whether labels for plant-based products are framed in terms of their health versus environmental benefits are associated with people’s liking and willingness to pay for the products. Green or environmental impact food labels are starting to be piloted by different retailers [12], but how consumers react to these may be dependent on several individual characteristics, suggesting there is not a “one-size-fits-all-approach”. Indeed, several recent studies have failed to document an overall effect of framing plant-based foods and diets in terms of their health versus environmental benefits [13,14,15]. This research therefore advances understandings of if and when the framing of plant-based food product labels can impact upon consumer liking and willingness to pay for plant-based products. It takes a novel approach in examining how an individual’s wellbeing at the time that they encounter a product could impact the persuasiveness of different types of label information. To the best of our knowledge, this is one of the first research studies to test an interaction between product label framings and individual wellbeing. The findings will have important implications concerning the effective marketing of plant-based food products.

### 1.1. Message Framings

Message framing describes the process of presenting information about a product, service, or issue in different ways to encourage more favourable reactions [16]. How the reasons why people should adopt plant-based diets are framed can influence peoples’ willingness to engage in this behaviour. Research has shown that framings which emphasize social norms [17], losses versus gains of (not) reducing meat consumption [18], and indulgent descriptors of plant-based products [19], amongst others, can all influence consumers preference for plant-based diets.

One avenue for message framing that is receiving increased attention in the behavioural science and sustainability literature involves emphasizing the co-benefits of sustainable actions. Research demonstrates that greater adoption of plant-based diets would not only benefit ecological sustainability, but also lead to a reduction in premature mortality rates [20], reduced levels of obesity [21], and chronic disease prevention [22]. Studies have shown that the expectation that plant-based diets tend to be healthier can act as a key driver for adopting more plant-based diets [23,24,25]. At the same time, uncertainty about nutritional sufficiency and negative beliefs about the protein content of plant-based foods have been shown to be barriers towards the adoption of plant-based diets [26,27].

Framing campaigns for plant-based products or diets in terms of their health, rather than environmental benefits, could therefore encourage the uptake of plant-based diets [28,29]. For instance, Wang et al. [30] have reported that Chinese consumers had greater preference for a plant-based burger when they were provided with positive nutritional information about the product. However, several other research studies have failed to demonstrate such a benefit [31,32]. For example, Vainio et al. [13] failed to show that employing a message frame which highlighted both the health and climate benefits of adopting plant-based diets was more effective at modifying behavioural intentions than a message that only emphasized the climate benefits in a sample of Finnish adults. Likewise, Wolstenholme et al. [14] tried to encourage students in the United Kingdom to reduce their consumption of red meat by telling them about the health benefits of reducing red meat consumption, the environmental benefits, or both. They found that all three messages were more effective than a control (no message) condition at reducing average reported consumption of red meat, but that there were no significant differences in the effectiveness of the three messaging conditions. Further, Lim et al. [15] sent young US adults text messages that either emphasized the health or environmental benefits of plant-based diets. They found no differences in the extent to which each message led to increases in consumption or intention to consume plant-based foods. This research therefore also hypothesized that labelling plant-based products in terms of their health versus environmental benefits alone would not be associated with subsequent differences in liking and willingness to pay for the products.

### 1.2. Wellbeing

Increasingly, research is documenting how engaging in sustainable behaviours, including adopting plant-based diets, is associated with greater personal wellbeing [33,34]. This effect is postulated to operate in both directions such that, as well as engagement in sustainable behaviours leading to improvements in wellbeing, the experience of high wellbeing can also prompt engagement in sustainable behaviours [35]. Indeed, several studies have now documented that inducing positive feelings can lead individuals to subsequently choose more sustainable behavioural options [36,37,38,39] as can the experience of higher levels of life satisfaction [40]. For example, Fröhlich et al. [41] ran an educational program for children aimed at getting them to consume in more environmentally friendly ways. They found that children’s levels of sustainable consumption at the end of the program were correlated with their situational emotions such that those experiencing high wellbeing reported more engagement in sustainable consumption whilst those experiencing negative emotions, such as boredom, reported less engagement in such actions. These positive effects of high wellbeing on pro-environmental behaviours have been theorised to either represent a desire of individuals to maintain their positive mood [37] or, in line with Fredrickson’s [42] broaden-and-build theory, the fact that positive emotions allow for more flexible patterns of thinking whereby people are more open to new behavioural options [43]. The present study therefore also expected higher levels of positive affect and life satisfaction to be associated with greater liking and willingness to pay for plant-based products.

It is not only positive emotions or life satisfaction that can influence sustainable choices. Negative emotions can also be successfully utilised to support interventions to promote sustainable behaviours [44]. When individuals are experiencing negative emotions, they are motivated to try to enhance their mood, and this can often be through engaging in pro-social and pro-environmental behaviours [37,45]. It is often written that individuals can experience a ‘warm glow’ from acting pro-environmentally, including reducing meat consumption [46]. Such effects may be especially likely when negative moods are linked to climate issues, such as climate anxiety or guilt [47,48].

However, other studies have documented the opposite effects, whereby the experience of high levels of negative affect is linked to reduced pro-environmental behaviours [43]. Potential explanations for negative effects of low mood on pro-environmental behaviour include the way that negative emotions may restrict thoughts and attention (making people less open to new behavioural options [42]) or make people more focused on the costs, rather than benefits, of specific actions [49]. It is therefore less clear whether higher levels of negative affect should be positively or negatively linked to participants’ liking and willingness to pay for plant-based products. In line with the theorising that negative affect highlights the costs of engaging in an action and limits openness to new options, and alongside the fact that the present research focuses on negative affect more broadly (rather than climate-specific emotions), the present study hypothesized that negative affect will be associated with lower liking and willingness to pay for the plant-based products overall.

### 1.3. An Interaction between Wellbeing and Message Framings

One of the reasons why information campaigns have been suggested to have limited effectiveness for increasing engagement in sustainable diets is because they often adopt a “one-size-fits-all approach” [50]. In doing so, they do not consider the audience’s specific characteristics or motivations for engaging in such behaviours, which may influence how consumers react to different message framings [51]. A handful of emerging studies are beginning to show that message framings are more effective when the framing employed is in line with the audience’s values [50,52], for example. This research tested whether personal wellbeing is another factor that can influence the effectiveness of different types of message framings. This potential interaction between an audience’s wellbeing and message framings may explain why studies have sometimes failed to find any differences in the effectiveness of framing plant-based diets in terms of their environmental versus health benefits.

The present study expected that higher life satisfaction and positive affect would lead to greater liking and willingness to pay for plant-based products in the environmental framing condition, given the research linking high wellbeing to pro-environmental behavioural choices [36,37,38,39,40]. In the health condition, however, this effect may be less pronounced, as several meta-analyses have failed to document that the experience of positive affect is significantly linked to engagement in health-related behaviours [53,54].

For negative affect, this research hypothesized the opposite pattern might occur. That is, when environmental frames are used, negative affect would be associated with lower liking and willingness to pay for plant-based products, in line with our theorising in the previous section around reduced openness to new behavioural options. When a health frame is used, however, the negative impact of negative affect on liking and willingness to pay may be weaker or even positive. This is because the health framing emphasizes the benefits that the product provides for the individual (improved health) to a greater extent. Therefore, it may be a more salient means of improving wellbeing for the individual, especially when the negative affect is not tied to environmental issues.

### 1.4. The Present Research

This research aims to develop understandings of the factors that can influence people’s liking and willingness to pay for plant-based products, focusing on the individual and interacting roles of food label framings and situational wellbeing. Whilst recent research has failed to document significant effects of message framings that emphasize the health over environmental benefits of engaging in plant-based diets on changes in dietary behaviours [13,14], the present research hypothesized that framings may have a bigger influence when considered in interaction with personal wellbeing. As discussed above, the research develops and builds upon existing work on message framing and wellbeing. The authors are not aware of any published research that has examined how situational wellbeing may interact with message framing to influence attitudes towards plant-based foods. If found, this interaction effect will inform marketers and retailers about the type of moods that need to be supported (e.g., within a store environment) to make people more susceptible to the specific message framing employed on plant-based food labels. Further, the small amount of existing research that has been conducted into the interaction between message framings and personal values, for example, has made use of written messages noting the different benefits of reducing meat consumption more broadly [50,51]. The present study, however, employs food labels to deliver the different message framings as, if environmental labels do become more commonplace, consumers are more likely to come across such labels than written campaign messages.

In line with the existing literature and theorising explored thus far, the following hypotheses were made:

**H1:**  *There will be no significant difference in the levels of liking and willingness to pay for plant-based products when they are framed in terms of their environmental versus health benefits.*

**H2:**  *Higher levels of life satisfaction and positive affect will be associated with greater liking and willingness to pay for plant-based products.*

**H3:**  *Higher levels of negative affect will be associated with lower liking and willingness to pay for plant-based products.*

**H4:**  *Higher levels of life satisfaction and positive affect will be associated with greater liking and willingness to pay for plant-based products when the product is framed in terms of its environmental (relative to health) benefits.*

**H5:**  *Higher levels of negative affect will be associated with lower liking and willingness to pay for plant-based products when the product is framed in terms of its environmental (relative to health) benefits.*

The hypotheses were tested across two online experiments. The two experiments employed largely the same design, materials, and measures, and were conducted to determine if the results would generalise across a university population (Study 1) and a non-student, adult sample (Study 2).

## 2. Study 1: University Sample

### 2.1. Materials and Method

#### 2.1.1. Participants

Participants were recruited using the online participant recruitment platform of a university situated in the southeast of England. One-hundred three individuals completed the study. The majority of these (79.5%) were full-time students of Psychology. The rest were either in full- or part-time employment within the university. Ninety participants were female and 13 were male. Participants mean age was 19 years old (*SD* = 2.57, min = 18, max = 33). The majority (69%) of participants described their ethnic origin as white. Whilst 68% of participants were undergraduate students, 29% had completed an undergraduate degree and 3% had completed a postgraduate degree. The majority (93%) of participants had an annual individual income of below £19,999. Participants who were students received one lab token in return for their participation.

#### 2.1.2. Experimental Design and Materials

**Figure 1:**
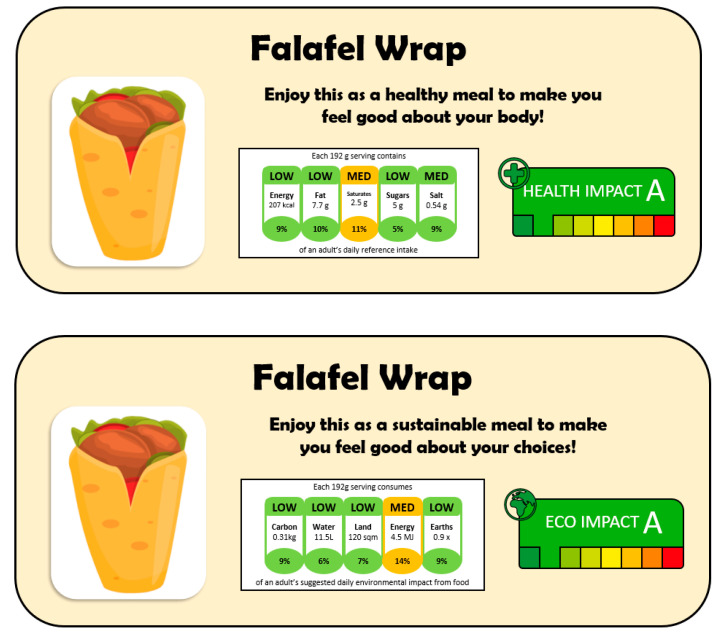
Example product label in the health (**top**) and environment (**bottom**) condition. Green impact ratings imply that the product is better for health/the environment.

Four hypothetical plant-based products were created for the sake of this study: bean burger, fruits smoothie, falafel wrap and tofu curry. For each product, two labels were created. The only difference between these two labels was that one contained health information whilst the other contained environmental information. In the health information condition, the product label included a ‘Health Impact A’ sticker, information on the nutritional content of the food, and the slogan “Enjoy this as a healthy meal to make you feel good about your body”. In the environmental information condition, the product label included an ‘Eco Impact A’ sticker, information on the environmental impacts of the food, and the slogan “Enjoy this as a sustainable meal to make you feel good about your choices”. All other details on the label (name, image, colour, size etc.) remained the same across the two conditions. See Figure 1 for an example of the two product labels for the falafel wrap. The numbers displayed in the nutrition and environmental impacts part of the label were based upon existing products in the same product category. Across both health and environmental labels, the product always scored an amber rating for one component within the label and a green rating on all the others.

A pilot study was conducted with 23 Psychology students to ensure that the hypothetical labels were projecting the desired framings. In this pilot, participants were presented with the four product labels (two environmental framing condition and two health framing condition). For each product, pilot test participants were asked to rate the extent to which they agreed that the product was (a) environmentally friendly, (b) good for health and wellbeing, (c) realistic, and (d) believable, on a scale from 0 (strongly disagree) to 10 (strongly agree). Paired samples *t*-tests revealed that the products displaying the environmental framing were rated as significantly more environmentally friendly than they were good for health and wellbeing, *t*(21) = 2.97, *p* < 0.01, d = 0.63. At the same time, the products displaying the health framing were more strongly rated as good for health and wellbeing than they were good for the environment, *t*(16) = −0.95, *p* = 0.18, d = 0.23. This difference was not statistically significant, but this may have been due to the small sample size. The mean scores for how realistic (overall *M* = 6.23, *SD* = 2.17) and believable (overall *M* = 6.5, *SD* = 2.16) participants considered each of the products to be, were above the scale mid-point suggesting suitable believability for our experiment.

At the start of the experiment, participants completed two measures of their subjective wellbeing. The first was the Satisfaction with Life Scale [55], which contains five items that individuals rate their agreement with on a scale of 1 (strongly disagree) to 7 (strongly agree). Example items include “The conditions of my life are excellent” and “I am satisfied with my life”. This scale therefore assessed each individual’s cognitive evaluation of the conditions of their own life and demonstrated excellent reliability in the present study (α = 0.91). The second scale was the 10-item international Positive and Negative Affect Schedule (PANAS) Short Form (I-PANAS-SF [56]). This assessed to what extent participants were experiencing a series of ten emotions at the time of the experiment, on a scale from 1 (very slightly or not at all) to 5 (extremely). Five of the items are positive emotions (α = 0.81) such as “alert” and “inspired” whilst five are negative emotions (α = 0.82) such as “upset” and “ashamed”.

The experiment employed a repeated measures design. All participants were exposed to the product labels for the four food products. Two of the labels included the health (nutrition) information whilst two included the environmental information. The products that were paired with each type of label (health or environment) were randomised across participants.

#### 2.1.3. Procedure

Participants completed the experiment online in a location of their choosing. The experiment was introduced as a study exploring people’s preferences for different food products. The study began with the completion of the subjective wellbeing measures, which were presented in a randomised order.

Participants were then told that a food and beverage company were in the process of designing a series of new products that they were hoping to bring to the market next year. As part of their market research, the company would like to get participants’ opinions on some of these new products. Participants were informed that on the following pages they would be presented with draft labels for four of the new products. The four product labels were presented one after the other. Two labels used the health framing whilst two the environmental framing. The order of product presentation was randomised across participants. For each product participants were asked five questions assessing their liking for the product, which they rated their agreement with on a scale of 1 (strongly disagree) to 7 (strongly agree). Example items included “This product is appealing to me” and “I would like to try this product”. They were also asked to indicate the maximum price in Great British Pounds that they would be willing to pay for the product.

After rating the four products participants were asked a few questions which were intended to act as controls within the analysis. They were asked to rate (a) how hungry they currently felt, (b) how often they typically try to eat a plant-based diet, (c) how socially desirable they thought it currently was to eat a plant-based diet and (d) the extent to which they agreed that eating an environmentally sustainable diet was better for their health. Following this, participants completed some basic demographic questions. The experiment ended by asking participants what they thought the experiment was trying to test. Participants responses tended to focus on “attitudes to plant-based diets”, “whether happy people care about the environment” and “how mood affects food choices”. No participants correctly guessed the specific aims of the study.

#### 2.1.4. Analysis Plan

The relationships between wellbeing, label framing condition and the two dependent variables (product liking and willingness to pay) were assessed using generalised estimating equations (GEE). GEE is an extension of regression analyses to account for repeated measurements within each participant [57]. In the present research, each participant provides responses for each of the four food labels. GEE also does not require the dependent variable to be normally distributed [58].

Demographic variables of age, gender, income, educational attainment, and employment status were controlled for within the GEE model. This followed existing work demonstrating that demographic variables were associated with attitudes towards and the consumption of more plant-based food options [23]. In addition, participants’ responses to the four questions assessing how hungry they were and their current beliefs and behaviours surrounding plant-based foods noted in the procedure section were controlled for.

Several main effects were then included within the model. These included the positive and negative affect subscale scores from the PANAS [56] along with participants’ total score on the Satisfaction with Life Scale [55]. Framing condition (environmental or health) was also included as a main effect within the model. Finally, interaction terms between framing condition and each of the three wellbeing variables were entered into the model. All analyses were conducted using SPSS [59].

### 2.2. Results and Discussion

The full output for the two GEE models assessing the predictors of product liking and willingness to pay are given in Table 1. Collinearity checks revealed there was not a problem of multicollinearity amongst the independent variables, with variable inflation factors (VIFs) all being less than 1.6. Further, Harman’s single factor test revealed that common method bias was unlikely to be present, with 19% of total variance explained by one common factor [60].

In line with H1, framing condition alone was not a significant predictor of levels of product liking or willingness to pay (H1 supported). Higher levels of life satisfaction were significantly associated with both greater liking for the plant-based products and willingness to pay more money for them, although positive affect was not (H2 partly supported). The negative affect scale did not prove to be a significant predictor of either outcome (H3 not supported), and there were no significant interaction terms between framing condition and the different wellbeing variables (H4 and H5 not supported).

Study 1 has therefore demonstrated that certain aspects of wellbeing (e.g., life satisfaction) are associated with consumers’ liking and willingness to pay for plant-based products. Whilst no main effects of framing condition were documented, in line with expectations, there were also no significant interaction terms. This was unexpected and means that currently it cannot be concluded that the reason for the lack of main effects surrounding messaging condition is because different messages are more effective for consumers with certain levels of situational wellbeing only.

## 3. Study 2: UK Adult Sample

Study 2 was conducted with the aim of testing whether the results of Study 1 would replicate in a non-university sample. The experimental design and analytical procedure were the same as in Study 1. Only two minor additions were made: The first was through the inclusion of a fifth plant-based product to allow us to include a control condition whereby the product label did *not* contain health or environmental information. This was to test whether including either type of label framing is better than not providing the health or environmental information at all. The second was through a second willingness to pay outcome variable. Study 1 required participants to openly state the maximum price they would pay for the products, but studies have shown that there can be discrepancies in willingness to pay judgements depending on whether open-ended or closed questions are employed [61,62]. Therefore, an additional closed, willingness to pay question was added in Study 2.

### 3.1. Materials and Method

#### 3.1.1. Participants

One hundred participants were recruited using the online survey recruitment platform, Prolific. An effort was made to recruit equal numbers of males and females as well as an equal spread of participants in the age groups of 25–40, 41–55 and 56+ years. Participants had to be based in the UK and not a student. They received £2 for their participation in the study. Fifty participants were male and fifty were female. Participants mean age was 49 years old (*SD* = 14.30, min = 25, max = 78). The majority (89%) of participants described their ethnic origin as white. Whilst 46% of the sample had completed an undergraduate degree, 16% had completed a postgraduate degree and 1% a doctorate. The median annual individual income bracket was £19,999–£39,999. The majority (60%) of the sample were employed, whilst 20% were retired and 20% unemployed.

#### 3.1.2. Experimental Design and Materials

The same four hypothetical plant-based products were used as in Study 1. A fifth product (cheesy vegan pasta pot) was added to allow for each participant to see two health, two environment, and one control framing product. The health and environment framing labels were the same as in Study 1. The control framing label did not include a health/environment impact sticker or information on the nutritional content/environmental impact of the food. Instead, it simply displayed the product name, image, a barcode, and the slogan “enjoy this as a nice meal to make you feel good.” The experiment employed the same repeated measures design as in Study 1.

All participants completed measures of their subjective wellbeing at the start of the experiment using the same two scales as in Study 1. In this study, scores on the Satisfaction with Life Scale (α = 0.93) as well as the negative (α = 0.86) and positive (α = 0.85) subscales of the PANAS showed good reliability.

#### 3.1.3. Procedure

The procedure in Study 2 was the same as in Study 1. Participants completed the experiment online in a location of their choosing and received the study link through the Prolific platform. They completed the measures of their wellbeing before being presented with the five product labels one after the other. Two labels used the health framing, two the environmental framing, and one the control framing. The order of product presentation, and which products were presented with which framing condition was randomised across participants.

For each product participants were asked the five questions assessing their liking for the product and the maximum price in pounds that they would be willing to pay for it. An additional question was also included whereby participants were asked to indicate how likely it would be, on a scale from 1 (very unlikely) to 5 (very likely), that they would pay a specific price for each product. The specific price stated was the average amount that participants in Study 1 said that they were willing to pay for each food product. For the new cheesy vegan pasta pot, the specific price was the overall mean across all food products from Study 1.

As in Study 1, the experiment ended with participants completing items that were intended to act as controls within the analysis and to assess their demographics. No participants correctly guessed the specific aims of the study.

#### 3.1.4. Analysis Plan

Comparison of liking and willingness to pay for the two messaging conditions, relative to the control condition, was assessed using a series of repeated measures ANOVA tests. The responses for the control condition were not included in the subsequent GEE models to allow for a direct replication of the statistical models employed in Study 1. All predictors in the GEE models were the same as those employed in Study 1. The only difference was that there were now three GEE models due to the inclusion of the further willingness to pay (closed) dependent variable.

### 3.2. Results and Discussion

#### 3.2.1. Effect of Messaging Conditions Relative to the Control

A series of repeated measures ANOVA tests (see Table 2) were performed to compare the effect of messaging condition on product liking and willingness to pay. Across all tests, Mauchly’s Test of Sphericity indicated that the assumption of sphericity had been violated and, therefore, a Greenhouse–Geisser correction was used.

There was not a significant effect of messaging condition on product liking, *F* (1.76, 174.56) = 0.95, *p* = 0.38. Similarly, for the willingness to pay (open) dependent variable, there was not a significant effect of messaging condition, *F* (1.51, 149.79) = 0.81, *p* = 0.42. However, for the willingness to pay (closed) dependent variable, there was a significant effect of messaging condition, *F* (1.82, 180.28) = 3.93, *p* = 0.025. Post-hoc pairwise comparisons with Bonferroni correction demonstrated that willingness to pay the stated price for the products was significantly higher in the environmental condition in comparison to the control condition (*p* = 0.018). None of the other conditions significantly differed from each other.

#### 3.2.2. GEE Replication

Collinearity checks revealed there was not a problem of multicollinearity amongst the independent variables, with variable inflation factors (VIFs) all being less than 1.5. Equally, Harman’s single factor test revealed that common method bias was unlikely to be present, with 23% of total variance explained by one common factor [60]. Study 2 (see Table 3 for outcome of GEE analysis) did not report any significant main effects of label framing condition on the outcome variables (H1 supported). Whilst life satisfaction scores were not significantly associated with any of the dependent variables, scores on the positive affect subscale of the PANAS were significantly and positively linked to all outcome variables (H2 partly supported).

Although negative affect scale did not display a main effect on any outcomes (H3 not supported), there was a significant interaction between framing condition and scores on the negative affect subscale of the PANAS for all outcome variables.

Graphical examination of this interaction alongside a GEE analysis (without the condition predictor or interaction terms) on the data for the two framing conditions separately demonstrated that, for product liking, there was no association with negative affect scores in the health framing condition (B = 0.05, SE = 0.17, *p* = 0.76). In the environmental framing condition, there was negative, although not quite statistically significant association between negative affect scores and product liking (B = −0.29, SE = 0.16, *p* = 0.07). Somewhat similar patterns emerged for the interactions between negative affect and willingness to pay variables. For the open-ended question, negative affect was not a statistically significant predictor in either framing condition. However, for the health framing condition the direction of the insignificant effect was positive (B = 0.007, SE = 0.04) whilst for the environmental condition it was negative (B = −0.006, SE = 0.04). For the closed-ended question, there was a positive, but not quite statistically significant, effect of negative affect scores in the health framing condition (B = 0.06, SE = 0.03, *p* = 0.08). In the environmental framing condition, there was a negative, non-significant association between negative affect scores and willingness to pay (B = −0.002, SE = 0.03, *p* = 0.95). Across these different outcomes there is a trend whereby negative affect has a more positive association with liking and willingness to pay in the health framing condition, but a more negative association with liking and willingness to pay in the environmental framing condition. H5 is therefore supported. There were no significant interactions between framing condition and the positive wellbeing variables (H4 not supported).

Study 2 has therefore supported the results of Study 1 by again showing no effect of label framing condition on the liking and willingness to pay variables. Also, somewhat in line with Study 1, it has shown that higher levels of positive affect are linked to greater willingness to pay for the products. Accordingly, high personal wellbeing does seem to encourage more favourable views of plant-based products. Unlike Study 1, Study 2 did find significant interactions between negative affect and framing condition across the three outcome variables. Accordingly, the research now documents some specific instances in which label framings may matter for forging product opinions. In particular, when negative affect is high, environmental framings may discourage consumption of plant-based products to a greater extent than health framings. Caution should be taken when drawing strong conclusions surrounding the interaction effect, however, given that the effects sizes appear to be small, and the effect was not replicated across both of our samples.

## 4. Overall Discussion

Given the need to drastically shift to more plant-based diets to help meet climate targets [1,2], this research sought to better understand the factors that can influence consumers’ liking and willingness to pay for plant-based products. Across two experiments, it tested the role of whether labels for plant-based products are framed in terms of their health versus environmental benefits and consumers’ situational wellbeing, along with whether an interaction between these variables exists. Several key themes have emerged from this work, which help to support and extend existing knowledge in this area. These are discussed along with practical implications and directions for future work below.

### 4.1. Key Findings and Implications

The first key finding from this work is that message framing on food labels alone does not appear to influence consumers’ liking and willingness to pay for plant-based products. Across both studies, framing had no main effect in the GEE analysis. In Study 2, comparing both types of framing with a control condition only revealed a small difference between the environmental and control conditions for the closed-ended willingness to pay question. There were no benefits to displaying the health information over the no information control across any of the outcome variables. This finding is in line with a handful of recent studies exploring the effects of message framings on willingness to adopt more plant-based diets. Such studies have documented a lack of significant effects of environmental versus health message framings using samples of Finnish adults [13], US adults [31,32], and university students in the UK [14]. This research adds to this evidence base by replicating the null finding within a UK university student sample and documenting it for the first time in a UK (non-university) adult sample. Accordingly, despite the evidence documenting that co-benefits to climate action, such as improved health and wellbeing, do exist [20,21,22], highlighting such benefits does not appear to improve attitudes and behavioural intentions towards eating plant-based foods.

Our study extends previous work in this area by employing product label framings, rather than short narrative descriptions surrounding the benefits of adopting plant-based diets (e.g., [13,14]). In the UK, schemes are being run to test the impact of food eco-labels, similar to those employed in our experiments, on the front of food packaging, and big-name brands such as Nestlé and Marks & Spencer have opted to take part [63]. In France, the government is aiming to make ecological labelling on food products mandatory [64]. If eco-labelling is to become compulsory, the current research suggests that it would not have large effects on consumer attitudes and behaviours, in comparison to showing the health information that is currently found on most food packaging. However, while adding environmental labelling may not be able to bring large, positive changes to consumer behaviour, it is not the case that the environmental label appears to cause lower liking or willingness to pay for the plant-based foods in comparison to the health framing. In other words, processes such as psychological reactance [65], whereby consumers will be motivated to avoid plant-based products if they feel labels are trying to manipulate their choices, are not evident within our findings. Accordingly, compulsory eco-labels should not reduce people’s liking and willingness to pay for plant-based foods.

The second key finding from this research concerns the positive effect of higher levels of personal wellbeing on liking and willingness to pay for the plant-based products. In Study 1, this effect was found through the significant, positive associations of subjective wellbeing and the outcome variables. In Study 2, this effect was found through the significant, positive associations of the positive affect subscale of the PANAs and the outcome variables. These findings support those documented in previous works showing that the experience of positive emotions or greater life satisfaction is linked to greater engagement in more sustainable behaviours such as recycling or reducing water consumption [37,38,39,40,41]. The present study demonstrates that such effects are also likely to occur for the pro-environmental behaviour of favouring plant-based foods.

A lot of the previous work into the relationships between wellbeing and sustainable behaviours have focused on the role of climate-related emotions. For example, inducing feeling of hope about humans’ ability to mitigate climate change, or guilt about human-caused environmental damages [44]. Others have argued that it is only a specific type of positive emotion that matters for promoting sustainable behaviours. For instance, Lange and Dewitte [66] failed to find that inducing positive or negative affect using video clips influenced participants’ choice to either leave the experiment early and induce an environmental cost or to stay in the experiment longer without the environmental cost. The authors proposed that the lack of significant effect in their case may reflect the fact that only certain types of emotions (e.g., awe) or certain types of pro-environmental actions (e.g., product choice rather than saving electricity) are subject to the positive effect of wellbeing on sustainable behaviour. This research has shown that non-climate specific emotions and life evaluations can also matter for promoting the uptake of plant-based diets. Accordingly, marketers and retailers may want to design store environments so as to induce higher levels of positive affect, in order to encourage positive attitudes and behaviours towards plant-based products.

On the other hand, levels of negative affect did not, on their own, influence liking or willingness to pay for the plant-based products. Existing theories have not been in agreement with whether negative emotions should hinder or support pro-environmental behaviours. For example, some emphasize a positive influence motivated by a desire to improve mood [37,44] while others highlight a negative influence through a limited openness to new options [42]. This research does not support either of these strings of argument.

However, in Study 2, levels of negative affect did interact with framing condition. The nature of these interactions across the three outcome measures demonstrated a trend whereby higher levels of negative affect were linked to a more positive association with liking and willingness to pay in the health framing condition, but a more negative association with liking and willingness to pay in the environmental framing condition. Such a pattern was most pronounced for product liking. This was in line with our hypotheses and suggests that high levels of negative affect may reduce liking and willingness to pay for plant-based products when their environmental benefits are made salient because of how negative affect reduces people’s openness to new behavioural options and increases focus on the costs of an action [42,49]. Plant-based products are often perceived as more costly [67]. When a health frame is used, however, the benefits of the food for the individual are more salient and hence, negative affect may encourage greater liking and willingness to pay as an individual may see the consumption of the plant-based food as a way of improving their wellbeing [37,45]. Practically, this finding suggests that adverts for plant-based foods which emphasize their environmental benefits should not be scheduled to appear following television or online content that is negative in emotional valence.

The question that remains is why these interaction effects between negative affect and framing were not statistically significant in Study 1. The Study 1 sample is dominated by young females on their way to completing a university education, while Study 2 includes a more equal spread of participants across genders and age groups. Research has documented that women tend to be more inclined to reduce their meat consumption than men [68], partly because of how eating meat is commonly considered as a masculine feature [69]. This greater inclination to adopt plant-based diets has been shown to be especially true for younger [70] and more highly educated individuals [23,71]. This more general inclination of the sample towards plant-based diets, based on existing demographic work, may have overridden any negative associations of negative affect when viewing the environmental labels, leading to a lack of significant interaction in Study 1.

### 4.2. Limitations and Future Directions

Employing an experiment presented advantages for controlling the design of the food labels, allowing for stronger inferences concerning the effects of the different label framings. Nevertheless, the research does have certain limitations and opens opportunities for future research. The use of the hypothetical food labels, for example, reduced the ecological validity of the experiment. If participants were shown real food products, perhaps physically in person, then their choices may have more accurately reflected their real-life purchase decisions. However, the use of real or physical products could also add further confounding variables such as the size or visual appeal of the product. Using cartoon images in our case helped to reduce the interference from these kinds of variables as participants could not see the actual product and so had to base their decisions on the label information provided. Future studies could aim to carry out natural experiments in collaboration with supermarkets and food retailers whereby the same product is labelled in different ways across stores. This would help to gather a more ecologically valid assessment of consumer reactions to different food labels while still controlling for the same product appearance, etc.

Qualitative work would also be a valuable addition to the current study. This paper has theorized around why, for example, negative affect could have differential associations with product liking and willingness to pay across messaging conditions. Conducting further interviews with participants following the experimental procedure would help to shed light on these theories and better understand their evaluation process when judging the different plant-based products. Based on our preliminary findings concerning the moderating role of negative affect in the association between message framings and liking/willingness to pay, further experiments could be conducted which manipulate participant’s levels of negative affect prior to exposure to different food products. Doing so would help to determine whether the influence of negative affect is causal or not. The results of such a study would have strong implications for the design of marketing campaigns for plant-based foods.

## 5. Conclusions

Given the need to transition to plant-based diets to meet environmental targets, this research sought to further explore the factors that influence consumers’ liking and willingness to pay for plant-based products. The results confirm findings from existing work showing that framing plant-based products in terms of their health versus environmental benefits does not have a significant impact on product liking or willingness to pay overall. Levels of consumer wellbeing, however, do seem to have a bigger impact, with higher levels of positive wellbeing (either life satisfaction or positive affect) being linked to greater liking and willingness to pay. Boosting consumer wellbeing may therefore be a means of encouraging the consumption of plant-based foods. In addition, Study 2 reported an interaction effect whereby levels of negative affect were differentially linked to liking and willingness to pay across the health and environmental framing conditions. This was such that, when labels employ a health framing, negative affect is positively linked to liking and willingness to pay. But when labels employ an environmental frame, negative affect is negatively linked to liking and willingness to pay. The findings therefore demonstrate the importance of considering the specific characteristics of the audience when designing the framings of food labels. When the intended audience is experiencing a certain degree of negative feelings, then labels which emphasize the health benefits may be more effective than those emphasizing environmental benefits.

## Figures and Tables

**Table 1:** Predictors of participants’ levels of product liking and willingness to pay in Study 1.

	DV = Product Liking			DV = Willingness to Pay		
	B ^a^	SE	*p*	B ^a^	SE	*p*
Age	0.23	0.19	0.22	0.03	0.05	0.55
Gender ^b^	0.25	1.05	0.81	−0.29	0.35	0.41
Education	1.20	1.03	0.24	−0.38	0.26	0.14
Income	0.18	0.86	0.83	−0.20	0.19	0.28
Employment ^c^	0.27	1.48	0.86	−0.30	0.32	0.35
How often plant-based	−2.21	0.47	**0.000**	−0.09	0.11	0.41
How socially desirable plant-based	−1.20	0.40	**0.003**	−0.24	0.12	0.06
Belief env sustain diet is healthy	0.14	0.42	0.73	0.01	0.08	0.85
Hunger	−0.15	0.19	0.43	−0.02	0.05	0.74
Framing condition ^d^	−0.98	3.42	0.77	−0.43	0.39	0.27
PANAS neg	0.12	0.18	0.59	0.04	0.04	0.08
PANAS pos	0.13	0.14	0.88	0.04	0.03	0.41
Life satisfaction	0.09	0.10	**0.006**	0.04	0.02	**0.009**
Framing × PANAS neg	−0.13	0.25	0.60	0.04	0.03	0.24
Framing × PANAS pos	−0.24	0.20	0.24	−0.03	0.02	0.21
Framing × Life satisfaction	0.17	0.12	0.16	0.01	0.01	0.33

Significant effects are highlighted in bold. Control variable outcomes are shown in the grey shaded area. ^a^ Unstandardized regression coefficient. ^b^ The reference group is male (vs. female). ^c^ The reference group is employed (vs. student). ^d^ The reference group is environmental information (vs. health information).

**Table 2:** Mean scores across messaging conditions for the three dependent variables in Study 2.

	Liking	Willingness to Pay (Open)	Willingness to Pay (Closed)
	M (SD)	M (SD)	M (SD)
Control	17.53 (8.95)	2.44 (1.22)	2.2 ^a^ (1.09)
Environmental framing	18.61 (7.15)	2.58 (0.90)	2.51 ^a^ (1.05)
Health framing	18.61 (7.51)	2.52 (0.92)	2.44 (1.07)

a indicates a statistically significant mean difference between conditions.

**Table 3:** Predictors of participants’ levels of product Liking and willingness to pay in Study 2.

	DV = Product Liking			DV = Willingness to Pay (Open)			DV = Willingness to Pay (Closed)		
	B ^a^	SE	*p*	B ^a^	SE	*p*	B ^a^	SE	*p*
Age	−0.10	0.04	0.01	−0.002	0.01	0.76	−0.01	0.07	0.11
Gender ^b^	0.75	0.88	0.39	0.36	0.13	**0.005**	0.29	0.15	0.06
Education	0.57	0.72	0.43	0.15	0.10	0.12	0.17	0.11	0.13
Income	−0.02	0.66	0.98	0.27	0.07	**0.000**	0.17	0.10	0.08
Employment (unemploy) ^c^	−0.39	1.31	0.77	−0.10	0.19	0.60	−0.01	0.25	0.97
Employment (retired) ^c^	−1.03	1.37	0.46	−0.33	0.20	0.09	−0.22	0.23	0.32
How often plant-based	−0.32	0.41	0.44	0.07	0.05	0.23	−0.02	0.07	0.81
How socially desirable plant-based	−1.27	0.62	**0.04**	−0.13	0.09	0.14	−0.16	0.10	0.14
Belief env sustain diet is healthy	1.47	0.54	**0.006**	0.05	0.06	0.39	−0.03	0.07	0.67
Hunger	0.28	0.16	0.08	0.03	0.02	0.20	0.04	0.03	0.16
Framing condition ^d^	2.03	3.47	0.56	−0.09	0.30	0.77	0.01	0.48	0.98
PANAS neg	−0.31	0.15	0.31	−0.03	0.04	0.72	−0.004	0.03	0.41
PANAS pos	0.45	0.16	**0.008**	0.04	0.02	**0.04**	0.07	0.02	**0.004**
Life satisfaction	0.16	0.10	0.11	0.001	0.01	0.94	0.007	0.02	0.96
Framing × PANAS neg	0.38	0.19	**0.04**	0.03	0.01	**0.02**	0.06	0.02	**0.009**
Framing × PANAS pos	−0.22	0.21	0.31	−0.01	0.02	0.82	−0.01	0.03	0.70
Framing × Life satisfaction	−0.07	0.11	0.54	−0.004	0.01	0.73	−0.01	0.01	0.34

Significant effects are highlighted in bold. Control variable outcomes are shown in the grey shaded area. ^a^ Unstandardized regression coefficient. ^b^ The reference group is male (vs. female). ^c^ The reference group is employed (vs. student). ^d^ The reference group is environmental information (vs. health information).

## Data Availability

The data presented in this study are openly available on the Open Science Framework at https://osf.io/g89dm/ (accessed on 1 August 2022).

## References

[1] Niemiec R., Jones M.S., Mertens A., Dillard C. (2021). The effectiveness of COVID-related message framing on public beliefs and behaviors related to plant-based diets. Appetite.

[2] Schiermeier Q. (2019). Eat less meat: UN climate-change report calls for change to human diet. Nature.

[3] Godfray H.C.J., Aveyard P., Garnett T., Hall J.W., Key T.J., Lorimer J., Pierrehumbert R.T., Scarborough P., Springmann M., Jebb S.A. (2018). Meat consumption, health, and the environment. Science.

[4] Reisinger A., Clark H., Cowie A.L., Emmet-Booth J., Gonzalez Fischer C., Herrero M., Howden M., Leahy S. (2021). How necessary and feasible are reductions of methane emissions from livestock to support stringent temperature goals?. Philos. Trans. R. Soc. A.

[5] Hilborn R., Banobi J., Hall S.J., Pucylowski T., Walsworth T.E. (2018). The environmental cost of animal source foods. Front. Ecol. Environ..

[6] Hemler E.C., Hu F.B. (2019). Plant-based diets for personal, population, and planetary health. Adv. Nutr..

[7] Willett W., Rockström J., Loken B., Springmann M., Lang T., Vermeulen S., Garnett T., Tilman D., DeClerck F., Wood A. (2019). Food in the Anthropocene: The EAT–Lancet Commission on healthy diets from sustainable food systems. Lancet.

[8] Xu X., Sharma P., Shu S., Lin T.-S., Ciais P., Tubiello F.N., Smith P., Campbell N., Jain A.K. (2021). Global greenhouse gas emissions from animal-based foods are twice those of plant-based foods. Nat. Food.

[9] Fresán U., Sabaté J. (2019). Vegetarian diets: Planetary health and its alignment with human health. Adv. Nutr..

[10] Sabaté J., Sranacharoenpong K., Harwatt H., Wien M., Soret S. (2015). The environmental cost of protein food choices. Public Health Nutr..

[11] Schenk P., Rössel J., Scholz M. (2018). Motivations and constraints of meat avoidance. Sustainability.

[12] Leach A.M., Emery K.A., Gephart J., Davis K.F., Erisman J.W., Leip A., Pace M.L., D’Odorico P., Carr J., Noll L.C. (2016). Environmental impact food labels combining carbon, nitrogen, and water footprints. Food Policy.

[13] Vainio A., Irz X., Hartikainen H. (2018). How effective are messages and their characteristics in changing behavioural intentions to substitute plant-based foods for red meat? The mediating role of prior beliefs. Appetite.

[14] Wolstenholme E., Poortinga W., Whitmarsh L. (2020). Two birds, one stone: The effectiveness of health and environmental messages to reduce meat consumption and encourage pro-environmental behavioral spillover. Front. Psychol..

[15] Lim T.J., Okine R.N., Kershaw J.C. (2021). Health- or Environment-Focused Text Messages as a Potential Strategy to Increase Plant-Based Eating among Young Adults: An Exploratory Study. Foods.

[16] Chong D., Druckman J.N. (2007). Framing theory. Annu. Rev. Polit. Sci..

[17] Stea S., Pickering G.J. (2019). Optimizing messaging to reduce red meat consumption. Environ. Commun..

[18] Carfora V., Pastore M., Catellani P. (2021). A cognitive-emotional model to explain message framing effects: Reducing meat consumption. Front. Psychol..

[19] Turnwald B.P., Boles D.Z., Crum A.J. (2017). Association between indulgent descriptions and vegetable consumption: Twisted carrots and dynamite beets. JAMA Intern. Med..

[20] Himics M., Giannakis E., Kushta J., Hristov J., Sahoo A., Perez-Dominguez I. (2022). Co-benefits of a flexitarian diet for air quality and human health in Europe. Ecol. Econ..

[21] Wang L., Cui S., Hu Y., O’Connor P., Gao B., Huang W., Zhang Y., Xu S. (2021). The co-benefits for food carbon footprint and overweight and obesity from dietary adjustments in China. J. Clean. Prod..

[22] Gibbs J., Cappuccio F.P. (2022). Plant-Based Dietary Patterns for Human and Planetary Health. Nutrients.

[23] Aschemann-Witzel J., Gantriis R.F., Fraga P., Perez-Cueto F.J. (2021). Plant-based food and protein trend from a business perspective: Markets, consumers, and the challenges and opportunities in the future. Crit. Rev. Food. Sci. Nutr..

[24] Circus V.E., Robison R. (2019). Exploring perceptions of sustainable proteins and meat attachment. Br. Food J..

[25] Contini C., Boncinelli F., Marone E., Scozzafava G., Casini L. (2020). Drivers of plant-based convenience foods consumption: Results of a multicomponent extension of the theory of planned behaviour. Food Qual. Prefer..

[26] Perez-Cueto F.J. (2020). Sustainability, health and consumer insights for plant-based food innovation. Int. J. Food Des..

[27] Reipurth M.F., Hørby L., Gregersen C.G., Bonke A., Cueto F.J.P. (2019). Barriers and facilitators towards adopting a more plant-based diet in a sample of Danish consumers. Food Qual. Prefer..

[28] Cordts A., Nitzko S., Spiller A. (2014). Consumer response to negative information on meat consumption in Germany. Int. Food Agribus. Manag. Rev..

[29] De Boer J., Schösler H., Boersema J.J. (2013). Climate change and meat eating: An inconvenient couple?. J. Environ. Psychol..

[30] Wang H., Chen Q., Zhu C., Bao J. (2022). Paying for the Greater Good?—What Information Matters for Beijing Consumers’ Willingness to Pay for Plant-Based Meat?. Foods.

[31] Whitley C.T., Gunderson R., Charters M. (2018). Public receptiveness to policies promoting plant-based diets: Framing effects and social psychological and structural influences. J. Environ. Policy Plan..

[32] Ye T., Mattila A.S. (2021). The effect of ad appeals and message framing on consumer responses to plant-based menu items. Int. J. Hosp. Manag..

[33] Milfont T.L., Satherley N., Osborne D., Wilson M.S., Sibley C.G. (2021). To meat, or not to meat: A longitudinal investigation of transitioning to and from plant-based diets. Appetite.

[34] Zawadzki S.J., Steg L., Bouman T. (2020). Meta-analytic evidence for a robust and positive association between individuals’ pro-environmental behaviors and their subjective wellbeing. Environ. Res. Lett..

[35] Zelenski J.M., Desrochers J.E. (2021). Can positive and self-transcendent emotions promote pro-environmental behavior?. Curr. Opin. Psychol..

[36] Bissing-Olson M.J., Iyer A., Fielding K.S., Zacher H. (2013). Relationships between daily affect and pro-environmental behavior at work: The moderating role of pro-environmental attitude. J. Organ. Behav..

[37] Chatelain G., Hille S.L., Sander D., Patel M., Hahnel U.J.J., Brosch T. (2018). Feel good, stay green: Positive affect promotes pro-environmental behaviors and mitigates compensatory “mental bookkeeping” effects. J. Environ. Psychol..

[38] Ibanez L., Moureau N., Roussel S. (2017). How do incidental emotions impact pro-environmental behavior? Evidence from the dictator game. J. Behav. Exp. Econ..

[39] Schneider C.R., Zaval L., Markowitz E.M. (2021). Positive emotions and climate change. Curr. Opin. Behav. Sci..

[40] Wang E., Kang N. (2019). Does life satisfaction matter for pro-environmental behavior? Empirical evidence from China General Social Survey. Qual. Quant..

[41] Fröhlich G., Sellmann D., Bogner F.X. (2013). The influence of situational emotions on the intention for sustainable consumer behaviour in a student-centred intervention. Environ. Educ. Res..

[42] Fredrickson B.L. (2001). The role of positive emotions in positive psychology: The broaden-and-build theory of positive emotions. Am. Psychol..

[43] Coelho F., Pereira M.C., Cruz L., Simões P., Barata E. (2017). Affect and the adoption of pro-environmental behaviour: A structural model. J. Environ. Psychol..

[44] Brosch T. (2021). Affect and emotions as drivers of climate change perception and action: A review. Curr. Opin. Behav. Sci..

[45] van Valkengoed A.M., Steg L. (2019). Meta-analyses of factors motivating climate change adaptation behaviour. Nat. Clim. Chang..

[46] Taufik D. (2018). Prospective “warm-glow” of reducing meat consumption in China: Emotional associations with intentions for meat consumption curtailment and consumption of meat substitutes. J. Environ. Psychol..

[47] Gao J., Zhao J., Wang J., Wang J. (2021). The influence mechanism of environmental anxiety on pro-environmental behaviour: The role of self-discrepancy. Int. J. Consum. Stud..

[48] Shipley N.J., van Riper C.J. (2021). Pride and guilt predict pro-environmental behavior: A meta-analysis of correlational and experimental evidence. J. Environ. Psychol..

[49] Yuen K.S., Lee T.M. (2003). Could mood state affect risk-taking decisions?. J. Affect. Disord..

[50] Verain M.C., Sijtsema S.J., Dagevos H., Antonides G. (2017). Attribute segmentation and communication effects on healthy and sustainable consumer diet intentions. Sustainability.

[51] Graham T., Abrahamse W. (2017). Communicating the climate impacts of meat consumption: The effect of values and message framing. Glob. Environ. Chang..

[52] Lagomarsino M., Lemarié L., Puntiroli M. (2020). When saving the planet is worth more than avoiding destruction: The importance of message framing when speaking to egoistic individuals. J. Bus. Res..

[53] Cameron D.S., Bertenshaw E.J., Sheeran P. (2015). The impact of positive affect on health cognitions and behaviours: A meta-analysis of the experimental evidence. Health Psychol. Rev..

[54] Ferrer R.A., Taber J.M., Sheeran P., Bryan A.D., Cameron L.D., Peters E., Klein W.M. (2020). The role of incidental affective states in appetitive risk behavior: A meta-analysis. Health Psychol..

[55] Diener E.D., Emmons R.A., Larsen R.J., Griffin S. (1985). The satisfaction with life scale. J. Personal. Assess..

[56] Thompson E.R. (2007). Development and validation of an internationally reliable short-form of the positive and negative affect schedule (PANAS). J. Cross-Cult. Psychol..

[57] Hubbard A.E., Ahern J., Fleischer N.L., Van der Laan M., Satariano S.A., Jewell N., Bruckner T., Satariano W.A. (2010). To GEE or not to GEE: Comparing population average and mixed models for estimating the associations between neighborhood risk factors and health. Epidemiology.

[58] Ballinger G.A. (2004). Using Generalized Estimating Equations for Longitudinal Data Analysis. Organ. Res. Methods.

[59] IBM Corp (2021). Released 2021. IBM SPSS Statistics for Windows, Version 28.0.

[60] Podsakoff P.M., MacKenzie S.B., Lee J.Y., Podsakoff N.P. (2003). Common method biases in behavioral research: A critical review of the literature and recommended remedies. J. Appl. Psychol..

[61] Frew E.J., Whynes D.K., Wolstenholme J.L. (2003). Eliciting willingness to pay: Comparing closed-ended with open-ended and payment scale formats. Med. Decis. Mak..

[62] Oerlemans L.A., Chan K.Y., Volschenk J. (2016). Willingness to pay for green electricity: A review of the contingent valuation literature and its sources of error. Renew. Sustain. Energy Rev..

[63] Howes O. Food Sustainability: New Eco Label Planned. https://www.which.co.uk/news/article/food-sustainability-environmental-scores-label-planned-a1b3t6S0otpc#:~:text=A%20pilot%20scheme%20to%20assess,friendly%20ways%20to%20produce%20food..

[64] FoodDrinkEurope Europe Targets Greenwashing and Eco-Labelling for Food. https://www.fooddrinkeurope.eu/europe-targets-greenwashing-and-eco-labelling-for-food/.

[65] Reynolds-Tylus T. (2019). Psychological reactance and persuasive health communication: A review of the literature. Front. Commun..

[66] Lange F., Dewitte S. (2020). Positive affect and pro-environmental behavior: A preregistered experiment. J. Econ. Psychol..

[67] Fehér A., Gazdecki M., Véha M., Szakály M., Szakály Z. (2020). A Comprehensive Review of the Benefits of and the Barriers to the Switch to a Plant-Based Diet. Sustainability.

[68] Graça J., Godinho C.A., Truninger M. (2019). Reducing meat consumption and following plant-based diets: Current evidence and future directions to inform integrated transitions. Trends Food Sci. Technol..

[69] Nakagawa S., Hart C. (2019). Where’s the beef? How masculinity exacerbates gender disparities in health behaviors. Socius.

[70] Clark L.F., Bogdan A.M. (2019). The role of plant-based foods in Canadian diets: A survey examining food choices, motivations and dietary identity. J. Food Prod. Mark..

[71] Bryant C.J. (2019). We can’t keep meating like this: Attitudes towards vegetarian and vegan diets in the United Kingdom. Sustainability.

